# Four-Point C2 Fixation for Unstable Atlas Fractures: Technical Note

**DOI:** 10.7759/cureus.75492

**Published:** 2024-12-10

**Authors:** Paul S Page, Seung Lee, William Clifton

**Affiliations:** 1 Neurological Surgery, Cleveland Clinic Foundation, Cleveland, USA; 2 Neurological Surgery, Mayo Clinic, Jacksonville, USA

**Keywords:** atlas fracture, biomechanics, cervical spine trauma, jefferson fracture, spine construct, spine trauma

## Abstract

Traumatic burst fractures of the atlas occur with axial loading of the cervical spine. Many of these injuries can be treated by nonsurgical management with external orthosis; however, cases with transverse ligament disruption or significant C1 lateral mass displacement require internal reduction and fixation. In patients with poor bone quality in the setting of osteoporosis or chronic illness, atlanto-axial fixation and reduction of the fracture can be a challenge, necessitating extension of fusion to the occiput, which significantly limits the range of motion. A 63-year-old man with a history of HIV, Parkinson’s disease, and osteoporosis presented with neck pain after a fall from sitting height. Imaging studies revealed an unstable C1 burst fracture with displacement of the C1 lateral masses. A novel four-point C2 fixation technique was used to reduce the fracture and provide stability to the construct in the setting of poor bone quality. To our knowledge, this is the first manuscript to describe the technique of four-point axis fixation for surgical reduction and fixation of an unstable atlas fracture.

## Introduction

Traumatic C1 burst fractures occur in 25% of craniocervical injuries and approximately 1% of all cervical spine injuries [1,2]. Surgical treatment of C1 fractures is reserved for patients with transverse ligament disruption, concomitant unstable cervical injuries, or significant C1 lateral mass displacement [3]. Open reduction and internal fixation of unstable atlas fractures can be a challenge due to the relatively small bony surfaces of the C1 lateral masses, which may be significantly displaced in unstable injuries [4]. Atlanto-axial fixation is the most common form of surgical treatment of unstable C1 burst fractures [3]. Anatomic variability in the C2 pars and pedicles may also pose a challenge in atlanto-axial fixation, requiring an extension of the fusion to the occiput, which carries higher non-union rates and a significantly limited postoperative range of motion [5]. C1 fractures in the elderly may also occur in the setting of osteoporosis and other chronic medical conditions, further increasing the risk of poor intraoperative fixation, postoperative complications, and non-union rates [6,7]. C2 laminar screws may augment the stability of a high-cervical construct; however, their use in conjunction with C2 pedicle/pars fixation has not been reported in a case of atlas fracture with lateral mass displacement [8]. In this technical note, we describe a technique for four-point C2 fixation in a patient with osteoporosis and Parkinson’s disease who presented with an unstable C1 fracture.

## Technical report

**Figure 1:**
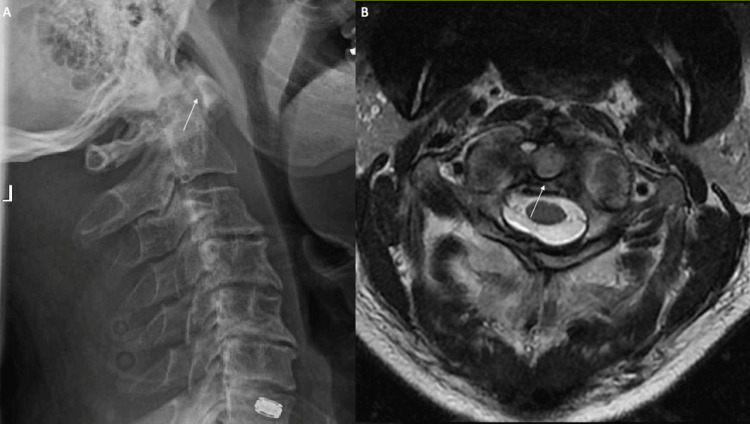
(A) Lateral cervical X-ray shows increased atlantodental interval of 5 mm, indicating instability (white arrow). (B) Axial T2 MRI demonstrated transverse ligament disruption (white arrow).

A 63-year-old male with a history of osteoporosis, HIV, and uncontrolled Parkinson’s disease presented to the emergency room 48 hours after sustaining a fall from a chair. He reported severe neck pain with head-turning. His neurologic exam revealed a moderate to severe resting tremor, but otherwise non-focal. He had midline neck tenderness at the base of his occiput and was not able to turn his head. Lateral X-ray showed an increased atlantodental interval (ADI) of 5 mm, indicating instability of the C1-2 complex (Figure 1).

**Figure 2:**
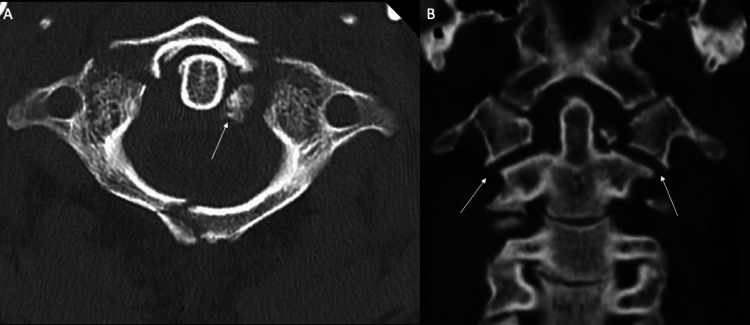
(A) Axial computed tomography shows displaced C1 fracture with transverse tubercle avulsion (white arrow). (B) Coronal images demonstrate approximately 7 mm of total lateral mass displacement (white arrows).

MRI did not show evidence of occipito-atlantal injury but did reveal transverse ligament disruption. A CT scan demonstrated a type 2 C1 burst fracture with displacement of the transverse tubercles bilaterally (Figure 2).

The patient was admitted and immediately placed in a cervical collar. After presenting the options of surgical vs. nonsurgical management, the patient elected to undergo open reduction and internal fixation. This was advised due to the patient’s history of repeated falls secondary to his Parkinson’s disease, increasing his risk for catastrophic neurologic injury if managed conservatively with external orthosis only.

**Figure 3:**
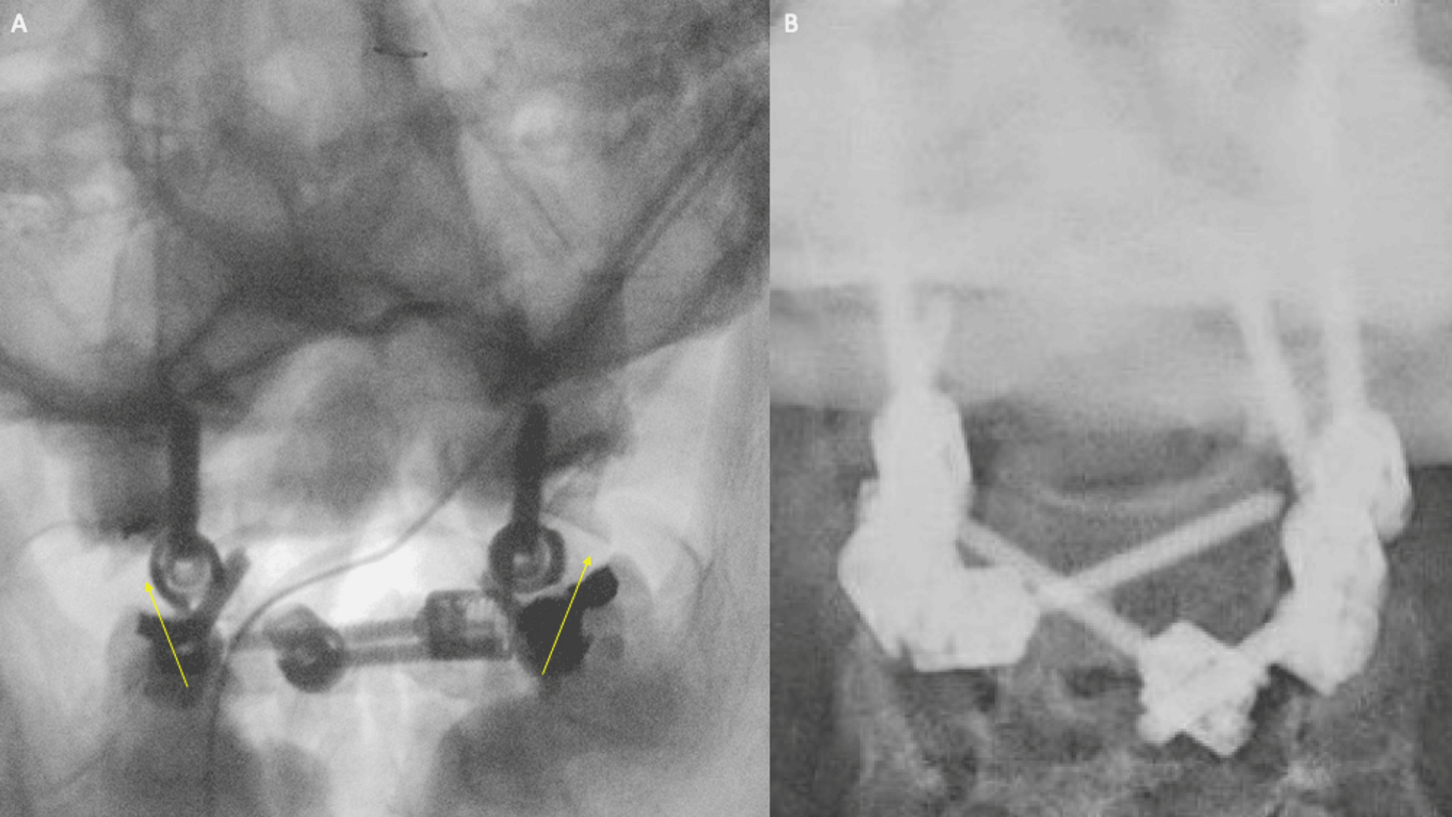
(A) Intraoperative X-ray after instrumentation shows significantly increased C1 lateral mass displacement compared to preoperative imaging (yellow arrows). (B) Post-reduction shows a connected construct with improved C1-2 lateral mass alignment.

Informed consent was obtained from the patient for a C1-2 fusion, with the possibility of extension to the occiput. After induction of general anesthesia, fiber-optic intubation was performed with the patient remaining in the cervical collar. Motor-evoked potentials (MEP) and somatosensory-evoked potentials (SSEP) were used to establish a baseline tracing for intraoperative monitoring. A Mayfield clamp was placed on the patient’s skull, and he was turned onto the operating table in a neutral position. During exposure, there was significant bruising seen in the nuchal ligament over the C1-2 level. The C2 lamina and C1 posterior arch were exposed, and the fractures of the C1 arch were dissected. The atlanto-axial membrane was disrupted with some hematoma present. Using a previously published technique, the C2 nerves were exposed bilaterally and sectioned at the preganglionic segments [9]. The C1 lateral masses were exposed and seen to be displaced to a much greater degree than preoperative imaging. The C2 pedicles were then cannulated bilaterally using direct visualization via a previously published technique [10]. The patient’s bone quality was found to be very poor. In addition, the right C2 pedicle was very thin, and a 3.5 x 26 mm screw was placed. On the left, a 4.0 x 26 mm screw was able to be placed. Bicortical C1 lateral mass screws were then placed using a drill, again with very poor bone quality. Bilaterally, 4.5 x 40 mm screws were placed. At this point, the C1 screw heads were at the same depth as the C2 screw heads; however, with substantial lateral mass displacement. Bilateral crossing 3.5 x 30 mm C2 laminar screws were then placed using the freehand technique according to a previously published method [11]. A cross connector was placed into the screw heads (Figure 3).

**Figure 4:**
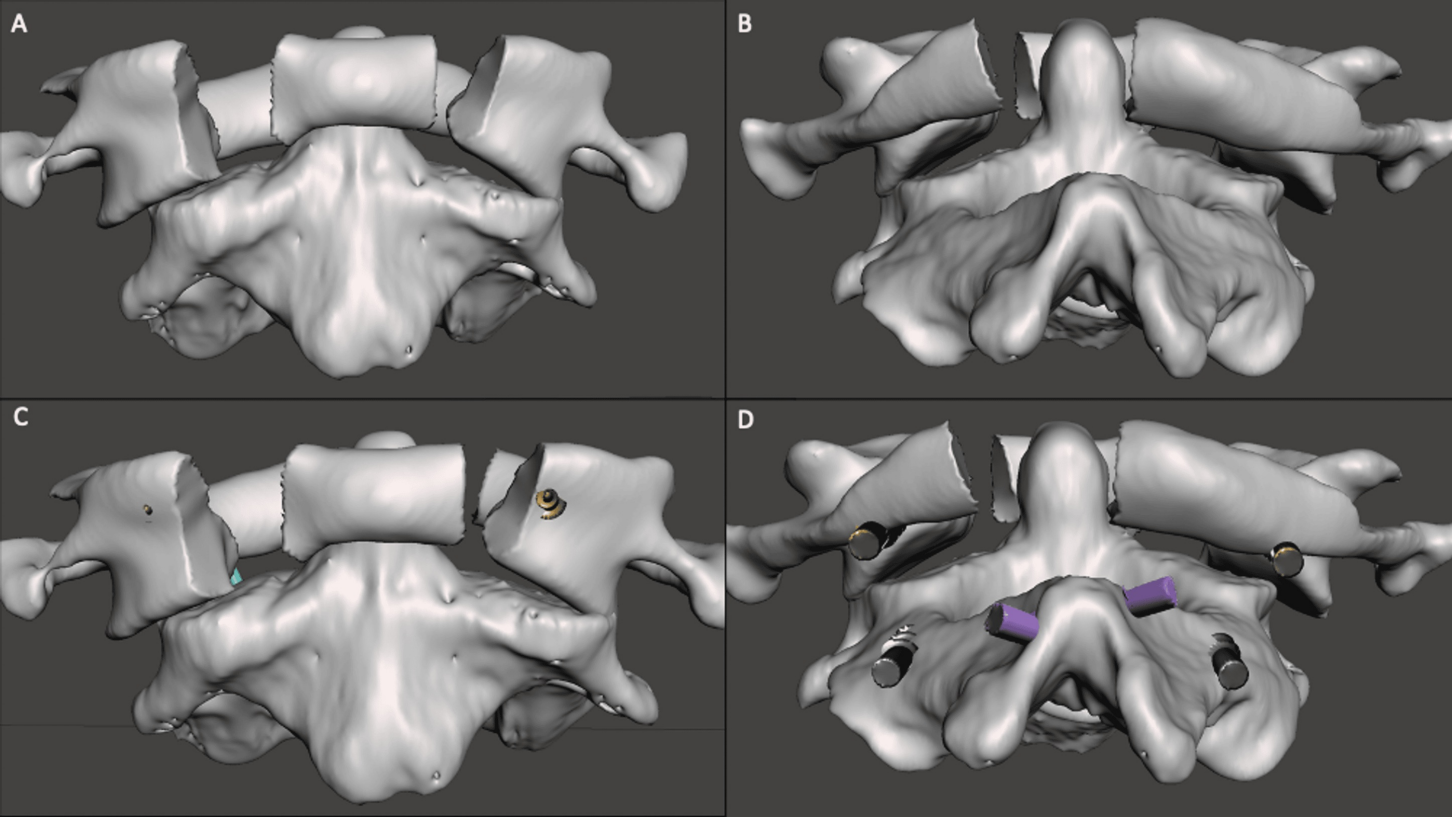
Three-dimensional simulation of C1 fracture and four-point C2 instrumentation. (A & B) C1 fracture with lateral displacement. (C & D) Instrumentation placement with C1 lateral mass screws and C2 pedicle and laminar screws in anatomic position.

A set screw was placed loosely to secure the cross connector. Bilateral rods were then placed into the C1 and C2 screw heads. The set screw to C1 was tightened, but the C2 pedicle screw was kept loose. The C1-2 rod was then connected to the lateral connector of the C2 laminar screw on each ipsilateral side (Figure 4).

**Figure 5:**
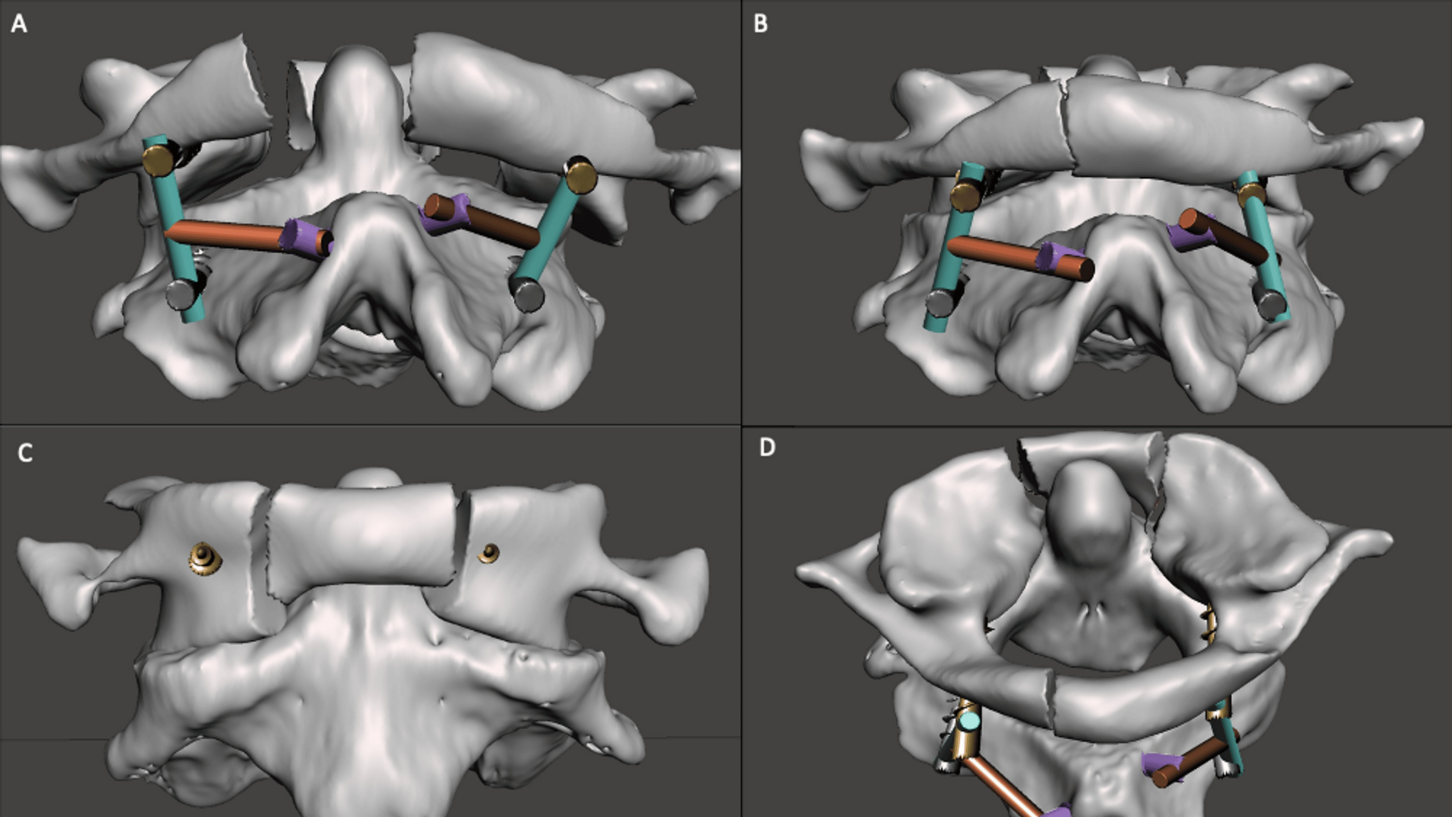
Stepwise reduction of C1 lateral masses using four-point C2 fixation. (A) Lateral connectors are placed in the screw heads of the C1 laminar screws bilaterally and connected to the C1-2 rods. (B) The rod is secured in the C1 lateral mass screw heads and loosened in the C2 pedicle screws and laminar screws. Gentle manual reduction is performed on the C1 lateral mass screw heads, allowing for the translation of the lateral connectors through the heads of the laminar screws. (C & D) The final construct is tightened with a secure reduction of the C1 lateral masses.

Using light manual reduction on the C1 lateral mass screw heads, the C1 lateral masses were reduced (Figure 5).

**Figure 6:**
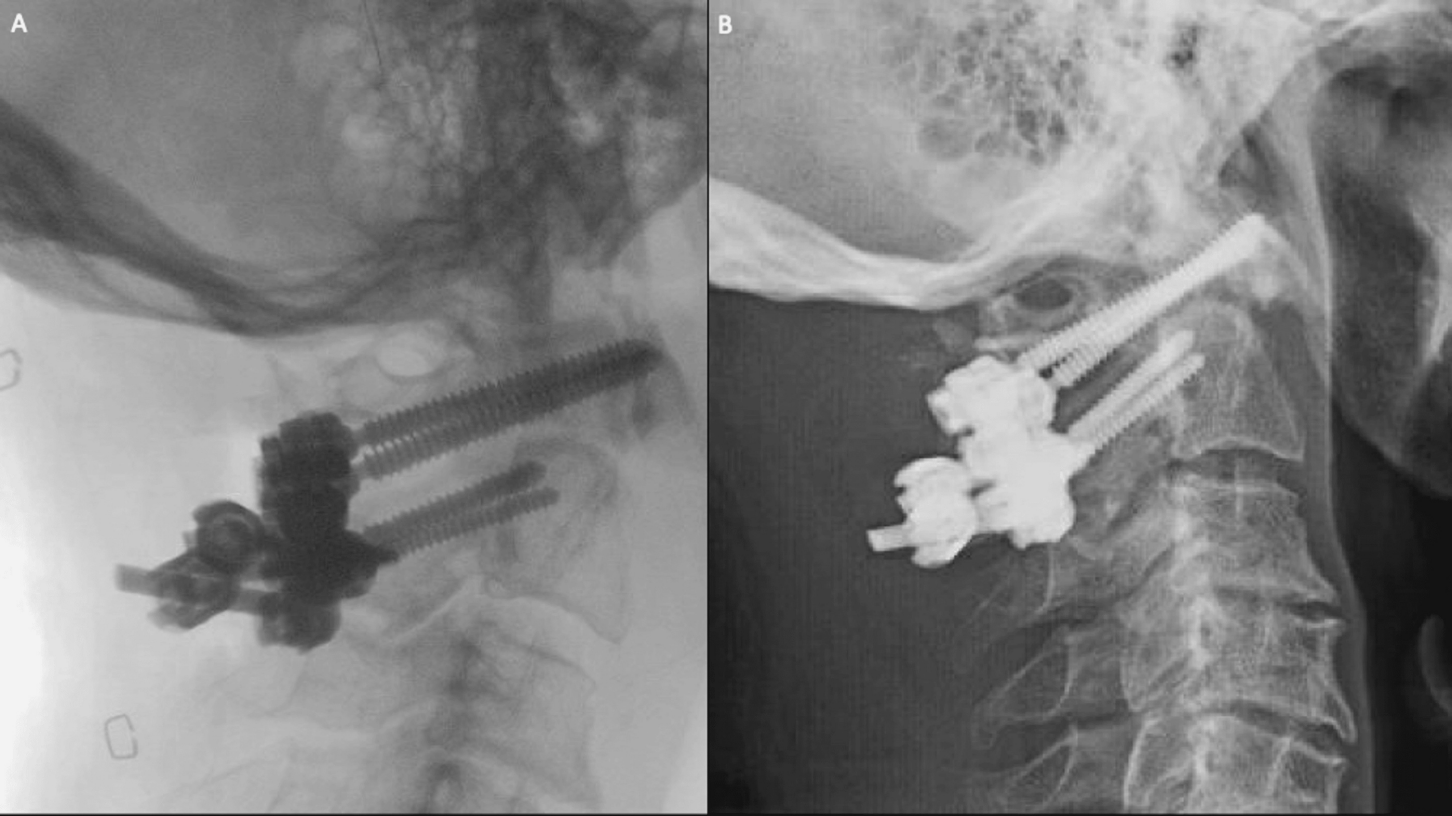
(A) Final intraoperative X-rays demonstrate improved atlantodental interval. (B) Eight-week postoperative X-rays showed no change in instrumentation, despite the patient’s non-compliance with external orthosis and Parkinsonian tremor.

The construct was then finally tightened. Intraoperative radiographs demonstrated excellent fracture reduction and position of the instrumentation (Figure 6).

Postoperatively, the patient had improvement in his preoperative neck pain and was ready for discharge on postoperative day two. At an eight-week follow-up, the patient had not been compliant with the collar and had a severe exacerbation of his Parkinson’s tremor. He continued to report no neck pain. Cervical films demonstrated no change from immediate postoperatively with stable placement of instrumentation.

## Discussion

This case highlights a novel intraoperative technique for C1 lateral mass reduction during internal fixation of an unstable C1 burst fracture. Previously reported techniques for intraoperative lateral mass reduction include the use of a cross connector, construct compression, and in-situ C1 fixation without atlantoaxial fusion [1,3,12]. Four-point C2 fixation has been previously reported in occipital-cervical constructs, increasing the biomechanical stability of occipital-cervical fixation [13]. The addition of C2 laminar screws in this case was paramount due to the patient’s severe osteoporosis and limited fixation of C2 due to anatomical constraints. This case also highlights the potential for increased intraoperative lateral mass displacement during instrumentation if the anterior arch is significantly compromised along with transverse ligament injury. Bicortical C1 lateral mass screws are preferred due to superior biomechanical strength than screws ending short of the ventral cortex [14]. Bicortical screws can increase the overall torque applied to the anterior portion of the screw during placement due to the long lever arm and cause increased lateral displacement of the fractured bone. In this scenario, reduction can be achieved by securing the C1 lateral mass screw to the C2 laminar screw via a cross connector. This connection acts as an anchor, allowing posterior coronal force to be applied without transference to the anterior fixation point of the lateral mass, as shown in this case. A potential technical challenge that may occur using this technique is planning bilateral laminar screw placement, and preoperative CT is necessary to measure the laminar thickness to ensure bilateral screw insertion without interaction, as previously published [11]. The authors prefer the utilization of a freehand technique for both C2 laminar and pedicle screw placement; however, the use of image guidance may also be employed for instrumentation planning and insertion [10,15]. The technique described may also be used with C2 pars fixation in patients with anatomy precluding the use of long pedicle screws [16]. When utilizing this technique, the surgeon should carefully evaluate the anatomy of the pars and the pedicles of C2 to optimize purchase.

C2 laminar screws alone have been reported for C1-2 fixation in fractures of the atlas with success; however, two-point axis fixation may not be sufficient in the setting of poor bone quality or severe instability [17-19]. The added stability of both pedicle and laminar screws in this case also obviated the need for extension to the occiput intraoperatively. Occipital-cervical fusions for high cervical injuries have been shown to have a higher incidence of dysphagia and reoperation rates, particularly in elderly patients compared with atlanto-axial constructs [20]. Aside from this, occipital cervical fusions are associated with a significantly greater loss of range of motion compared with atlanto-axial constructs. Although sometimes necessary, avoidance of occiput extension is preferred in the surgical treatment of axis fractures. The addition of biomechanical support via four-point fixation of C2 may prevent the need for inclusion of the occiput into atlanto-axial constructs in cases of poor bone quality.

## Conclusions

Unstable fractures of the atlas carry significant technical challenges. This technical note highlights a novel technique for including laminar screws into a C1-2 instrumented construct for added biomechanical stability as well as lateral mass reduction in the setting of intraoperative displacement. This technique can be safely used as a salvage procedure in patients with severe osteoporosis or other medical conditions that place the patient at high risk for a nonunion. Through the use of unique construct designs such as this, the surgeon can avoid the inclusion of additional levels and occipital cervical fixation and its associated morbidity.
